# A photofunctional bottom-up bis(dipyrrinato)zinc(II) complex nanosheet

**DOI:** 10.1038/ncomms7713

**Published:** 2015-04-02

**Authors:** Ryota Sakamoto, Ken Hoshiko, Qian Liu, Toshiki Yagi, Tatsuhiro Nagayama, Shinpei Kusaka, Mizuho Tsuchiya, Yasutaka Kitagawa, Wai-Yeung Wong, Hiroshi Nishihara

**Affiliations:** 1 Department of Chemistry, Graduate School of Science, The University of Tokyo, 7-3-1, Hongo, Bunkyo-ku, Tokyo 113-0033, Japan; 2 Institute of Molecular Functional Materials, Department of Chemistry and Partner State Key Laboratory of Environmental and Biological Analysis, Hong Kong Baptist University, Waterloo Road, Hong Kong, China; 3 Division of Chemical Engineering, Department of Materials Engineering Science, Graduate School of Engineering Science, Osaka University, 1-3, Machikaneyama, Toyonaka, Osaka 560-8531, Japan; 4 Department of Chemistry, Graduate School of Science, Osaka University, 1-1, Machikaneyama, Toyonaka, Osaka 560-0043, Japan; 5 HKBU Institute of Research and Continuing Education, Shenzhen Virtual University Park, Shenzhen 518057, China

## Abstract

Two-dimensional polymeric nanosheets have recently gained much attention, particularly top-down nanosheets such as graphene and metal chalcogenides originating from bulk-layered mother materials. Although molecule-based bottom-up nanosheets manufactured directly from molecular components can exhibit greater structural diversity than top-down nanosheets, the bottom-up nanosheets reported thus far lack useful functionalities. Here we show the design and synthesis of a bottom-up nanosheet featuring a photoactive bis(dipyrrinato)zinc(II) complex motif. A liquid/liquid interfacial synthesis between a three-way dipyrrin ligand and zinc(II) ions results in a multi-layer nanosheet, whereas an air/liquid interfacial reaction produces a single-layer or few-layer nanosheet with domain sizes of >10 μm on one side. The bis(dipyrrinato)zinc(II) metal complex nanosheet is easy to deposit on various substrates using the Langmuir–Schäfer process. The nanosheet deposited on a transparent SnO_2_ electrode functions as a photoanode in a photoelectric conversion system, and is thus the first photofunctional bottom-up nanosheet.

## Abstract (web summary)

The creation of functional 2D bottom-up nanosheets woven from molecular components remains a large challenge. Here, a bottom-up nanosheet featuring a photofunctional bis(dipyrrinato)zinc(II) complex motif is synthesized using interfacial syntheses, enabling a photoelectric conversion system.

## Graphical abstract

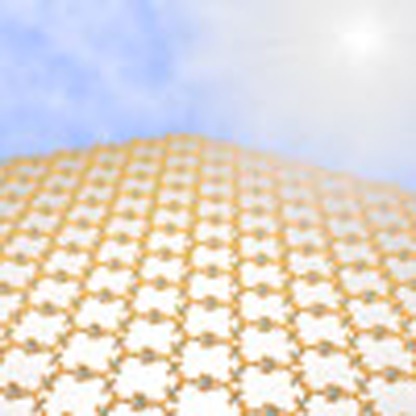

Nanosheets with two-dimensional polymeric structures have recently attracted significant attention. Graphene, a prominent nanosheet with various attractive properties, has been investigated extensively for applications in electronics^1^,^2^,^3^, photonics^4^ and spintronics^5^,^6^. The broad attention and expected utility of graphene have stimulated research into other nanosheets, such as metal oxides^7^,^8^,^9^,^10^,^11^, metal sulfides^12^,^13^,^14^,^15^ and metal hydroxides^16^,^17^,^18^,^19^,^20^, which also exhibit desirable and useful properties (for example, semiconductivity^10^,^15^ and ferroelectricity^8^,^14^). These nanosheets originate from bulk-layered crystalline mother materials (that is, top-down nanosheets).

Another type of nanosheets, namely molecule-based bottom-up nanosheets, is emerging. This series of nanosheets is fabricated directly from atomic, ionic and molecular components. The concept was proposed nearly a century ago, although it has only recently been realized^21^. For example, Schlüter and King created independently single-layer cycloaddition-induced anthracene^22^,^23^,^24^,^25^ nanosheets that featured carbon–carbon covalent bonds. 2,2′:6′,2′′-Terpyridine/metal complex nanosheets based on a metal–ligand coordination linkage were reported by Schlüter^26^,^27^. Frauenrath synthesized a carbon-rich nanosheet using carbonization of an amphiphilic hexayne molecule^28^. Other examples include surface metal-organic frameworks^29^,^30^, surface covalent-organic frameworks^31^,^32^,^33^,^34^, metal-surface-mediated monolayer formation in the vacuum phase^35^,^36^,^37^,^38^,^39^, and single-layer or few-layer metal-organic frameworks and covalent-organic frameworks delaminated from the crystal phase^40^,^41^,^42^,^43^,^44^,^45^,^46^. A significant advantage of the bottom-up synthesis is that structures can be customized through the selection of components (for example, metal ions and organic ligand molecules). Therefore, the bottom-up approach may broaden the diversity and utility of nanosheets. Although previous reports on bottom-up nanosheets have concentrated on the fabrication and analysis of various two-dimensional structures, no functionality has yet been demonstrated.

Given this background, we have sought to create functional bottom-up nanosheets, including an electrically conductive nickel bis(dithiolene) nanosheet^47^,^48^,^49^. The present work describes a bis(dipyrrinato)zinc(II) complex nanosheet synthesized from a three-way dipyrrin ligand and zinc(II) acetate. The spontaneous and reversible coordination of dipyrrin ligands with metal ions^50^,^51^ makes them suitable as building blocks for bottom-up nanosheets. The bis(dipyrrinato)zinc(II) complex motif acts not only as a connecting point but also as a photofunctional moiety: it has a strong absorption in the visible and near-infrared region (tunable by substituents)^52^,^53^,^54^. In addition to the synthesis and identification of the bis(dipyrrinato)zinc(II) complex nanosheet, we report its large domain size (sides of >10 μm), guest inclusion, stepwise layering and photoelectric conversion ability.

## Results

### Synthesis and morphology of multi-layer N1

**Figure 1:**
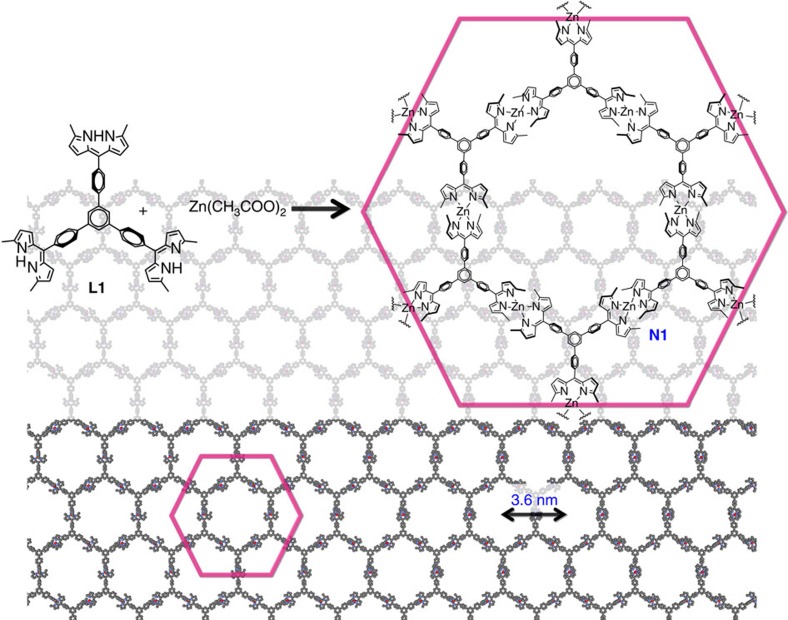
Bottom-up nanosheet of the present work. Chemical structures of three-way dipyrrin ligand molecule **L1** and bis(dipyrrinato)zinc(II) complex nanosheet **N1**.

**Figure 2:**
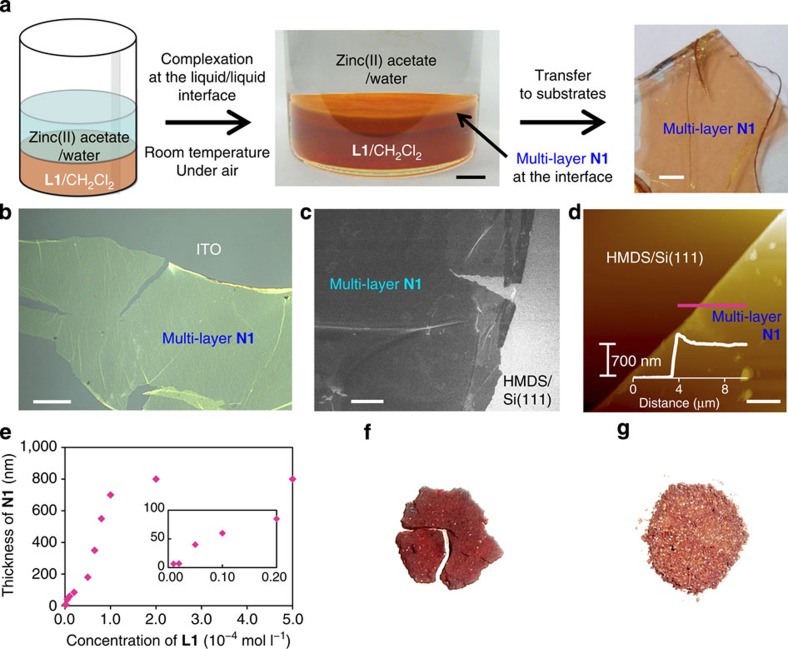
Synthesis and microscopic analysis of multi-layer N1. (**a**) Schematic illustration and photographs of the liquid/liquid interfacial synthesis and multi-layer **N1** transferred onto an ITO substrate. Scale bars, 5 and 1 mm, respectively. (**b**) Optical microscopic image on an ITO substrate. Scale bar, 50 μm. (**c**) Field-emission scanning electron microscopic (FE-SEM) image on HMDS/Si(111). Scale bar, 20 μm. (**d**) Atomic force microscopic image on HMDS/Si(111) and its cross-section analysis along the magenta line. Scale bar, 5 μm. (**e**) Control of the thickness based on the concentration of **L1** in the liquid/liquid interfacial synthesis. The inset shows a close-up of the low concentration region. Reaction time, 4 days. Temperature, room temperature. Container, cylindrical glass vial with a diameter of 3.2 cm. Volume of the upper (aqueous) layer, 20 ml. Volume of the lower (dichloromethane) layer, 10 ml. Concentration of zinc(II) acetate in the upper layer, 0.05 mol l^−1^. (**f**,**g**) Photographs of the products of single-phase reactions in dichloromethane at room temperature and in *N*,*N*-dimethylformamide at 105 °C, respectively.

Figure 1 depicts bis(dipyrrinato)zinc(II) complex nanosheet **N1** synthesized from three-way dipyrrin ligand **L1** and zinc(II) acetate. To verify the macroscopic formation of **N1**, the first process considered was a liquid/liquid interfacial synthesis^47^ using an aqueous zinc(II) acetate solution (upper layer, 5.0 × 10^−2^ mol l^−1^) and a dichloromethane solution of **L1** (lower layer, 1.0 × 10^−4^ mol l^−1^; Fig. 2a). A spontaneous reaction at room temperature led to the generation of multi-layer **N1** at the water/oil interface, which appeared as an orange film. Multi-layer **N1** may be transferred from the interface onto various substrates. For example, on an indium tin oxide (ITO) substrate it appears as a transparent film (Fig. 2a). Multi-layer **N1** is not soluble in either water or organic solvent, reflecting the polymeric structure proposed in Fig. 1. Optical and scanning electron microscopic images reveal a uniform, flat, film-like morphology (Fig. 2b,c). The presence of cracks and wrinkles also indicates the sheet structure. The typical thickness of multi-layer **N1** on a silicon(111) substrate modified with 1,1,1,3,3,3-hexamethyldisilazane (HMDS/Si(111)) was observed to be 700 nm by atomic force microscopy (AFM, Fig. 2d), which corresponds to ~580 layers considering the thickness of single-layer **N1** (*vide infra*). The thickness of **N1** may be controlled by the concentration of **L1** in dichloromethane and may span 6–800 nm (5–670 layers) using **L1** solutions with concentrations of 1.0 × 10^−6^ to 5.0 × 10^−4^ mol l^−1^ (Fig. 2e). We note that the liquid/liquid interfacial synthesis is essential for the synthesis of multi-layer **N1**. A conventional single-phase synthesis, performed in either dichloromethane at room temperature or *N*,*N*-dimethylformamide at 105 °C, resulted in a solid material far from a film texture (Fig. 2f,g). In fact, its disordered structure was verified by X-ray photoelectron spectroscopy (XPS, *vide infra*).

### Synthesis of single-layer or few-layer N1

**Figure 3:**
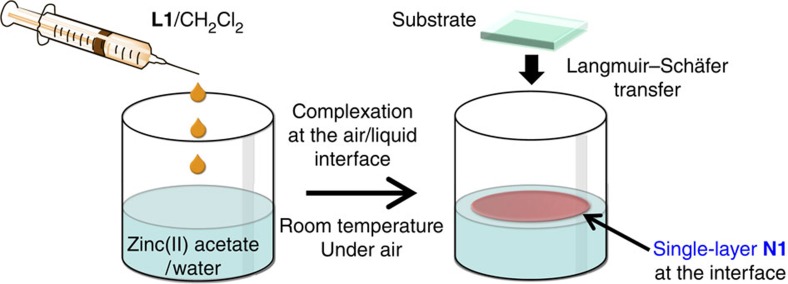
Synthesis of single-layer N1. Schematic illustration of the air/liquid interfacial synthesis and transfer process.

Next, we synthesized single-layer **N1**. Figure 3a shows the fabrication procedure, an air/liquid interfacial synthesis^47^,^48^,^49^. A very tiny amount (5 μl) of a dilute dichloromethane solution of **L1** (7.4 × 10^−5^ mol l^−1^) was gently dropped onto the surface of an aqueous phase containing zinc(II) acetate (5.0 × 10^−2^ mol l^−1^) at ambient temperature. After prompt evaporation of dichloromethane, spontaneous air/water interfacial complexation occurred between **L1** and zinc(II) ions, and single-layer **N1** was produced at the interface. The resultant single-layer **N1** was almost invisible to the naked eye but could be transferred onto various substrates via horizontal deposition known as the Langmuir–Schäfer method (Fig. 3). We note that few-layer **N1** was also synthesized by increasing the dichloromethane solution of **L1** to 20 μl. The single-layer or few-layer **N1** on a flat substrate was then subjected to a series of analyses.

### XPS of N1

**Figure 4:**
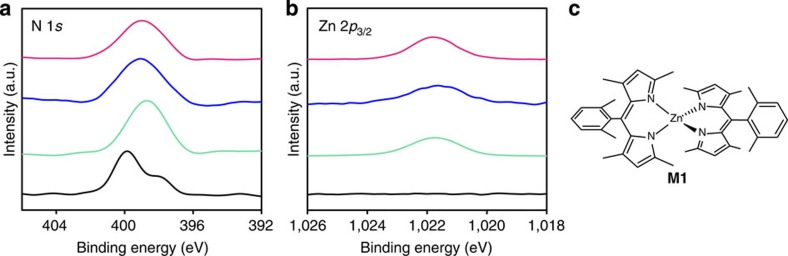
XPS for N1 and referential compounds. (**a**) Narrow-scan XPS focusing on the N 1*s* region. (**b**) Focusing on the Zn 2*p*3/2 region. Legend: **L1** on HMDS/Si(111) (black), **M1** on adhesive carbon tape (turquoise), multi-layer **N1** on HOPG (blue) and single-layer **N1** on HMDS/Si(111) (magenta). The intensity is proportional to the element abundance: the original signal is standardized using the photoionization cross-section of each element. (**c**) Mononuclear bis(dipyrrinato)zinc(II) reference complex **M1**.

XPS was conducted for single-layer **N1** on HMDS/Si(111) to assess its constituent elements (N and Zn) and their bonding properties (Fig. 4a,b). For reference, dipyrrin ligand **L1**, mononuclear bis(dipyrrinato)zinc(II) complex **M1** (Fig. 4c) and multi-layer **N1** deposited on highly ordered pyrolitic graphite (HOPG) were also subjected to XPS. Ligand **L1** did not show a Zn 2*p*_3/2_ peak, whereas the peak was present at similar binding energies in the spectra of **M1**, multi-layer **N1** and single-layer **N1** (1021.8, 1021.6 and 1021.8 eV, respectively). The spectrum of **L1** features two N 1*s* peaks (400.0 and 398.4 eV), assignable to pyrrolic and iminic nitrogen atoms, respectively^55^, whereas **M1** shows a single N 1*s* peak at 398.8 eV, which originates from its coordination to the zinc(II) centre, which makes the two nitrogen atoms equivalent to one another. Both multi-layer **N1** and single-layer **N1** displayed a single peak for N 1*s* at almost the same binding energy as **M1** (399.0 eV). In addition, the abundance ratio calculated from the peak area corrected using the photoionization cross-section is consistent with the ideal value of N:Zn=4:1 (80.6:19.4, 80.7:19.3 and 79.1:20.9 for **M1**, multi-layer **N1** and single-layer **N1**, respectively). These findings indicate that the bis(dipyrrinato)zinc(II) complex motif formed quantitatively in the nanosheet. We note that coordination of H_2_O molecule(s) to the zinc(II) centre to form a square–pyramidal or *trans*-octahedral coordination sphere may be excluded by the fact that multi-layer **N1** on oxygen-free HOPG lacked an O 1*s* peak in XPS (Supplementary Fig. 1). Also noteworthy is that the products of the single-phase reactions (Fig. 2f,g) exhibited nitrogen abundancies in excess of the ideal stoichiometric ratio (N:Zn=5.4:1 and 5.8:1, Supplementary Fig. 2). This result indicates that the single-phase reaction products leave uncoordinating dipyrrin moieties, thereby possessing disordered structures.

### AFM of single-layer or few-layer N1

**Figure 5:**
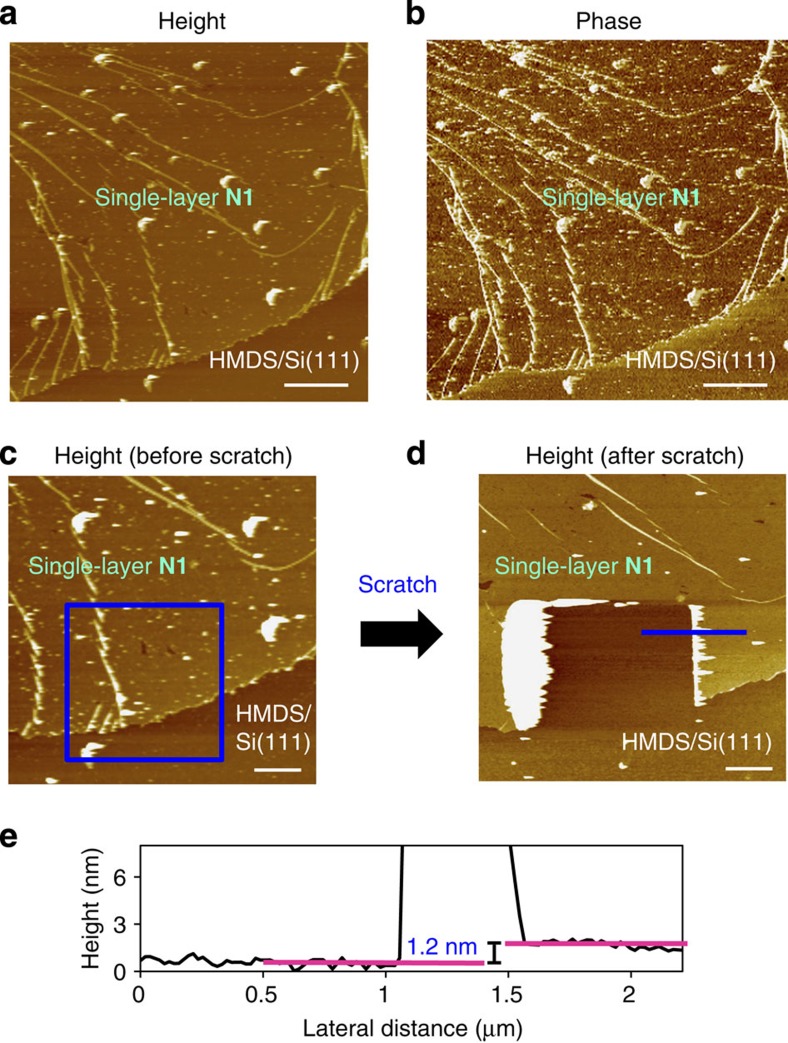
Atomic force microscopy for single-layer N1. (**a**,**b**) Height and phase images on HMDS/Si(111). Scale bar, 2 μm. (**c**,**d**) Height images before and after a scratch by the AFM tip. The blue square indicates the scratched region. Scale bar, 1 μm. (**e**) Cross-section analysis at one of the steps in the scratched region (shown as a blue line in **d**).

The AFM analysis revealed the flat sheet texture and morphology of single-layer **N1**. Figure 5a,b shows the height and phase images, respectively, of single-layer **N1** on an HMDS/Si(111) substrate. The phase image clearly distinguishes single-layer **N1** and the bare substrate: the nanosheet possesses a phase value that is lower by 2.4°. The height image shows a domain with one side >10-μm long, which is a noteworthy size for bottom-up nanosheets^29^,^30^,^31^,^32^,^33^,^34^,^35^,^36^,^37^,^38^,^39^. To confirm the single-layer nature of the sheet, part of **N1** was scratched with the AFM tip at a force of 8.6 × 10^2^ nN. Figure 5c,d shows the height images before and after scratching, and the scratched region is highlighted with a blue square. This treatment resulted in the removal of **N1**, leaving an intact HMDS/Si(111) surface. A cross-section analysis traversing one of the scratched edges demonstrated that **N1** was 1.2-nm thick (Fig. 5e), which is consistent with the size of the bis(dipyrrinato)zinc(II) complex motif. A higher-force scratch (1.2 × 10^4^ nN) resulted in a more drastic change in the AFM image (Supplementary Fig. 3). In this case, both single-layer **N1** and the HMDS/Si(111) surface were destroyed.

Few-layer **N1** with larger domain sizes was fabricated by increasing the amount of **L1** (Supplementary Fig. 4). The phase image is flat, whereas the topological image reveals steps and divided domains. This result indicates that the scanned area was completely covered with few-layer **N1**. The wrinkles in the layers may be evidence of the sheet structure and were removed by thermal annealing at 120°C (Supplementary Fig. 5).

### In-plane periodicity

Scanning tunnelling microscopy (STM) revealed that single-layer **N1** on HOPG exhibited a moiré pattern composed of two lattices: one is the in-plane hexagonal periodicity of **N1** and the other is derived from HOPG (Supplementary Fig. 6). The in-plane periodicity was also confirmed by selected area electron diffractions (SAEDs) in transmission electron microscopy for multi-layer **N1** (Supplementary Fig. 7). They represent two sets of hexagonal diffractions, which are consistent with in-plane diffraction patterns reproduced from crystal lattices comprising piles of single-layer **N1**, which were optimized using a molecular mechanics calculation (Supplementary Fig. 8; Supplementary Information). This series of analyses ensures the hexagonal in-plane periodicity of **N1**.

### Optical properties and layering

**Figure 6:**
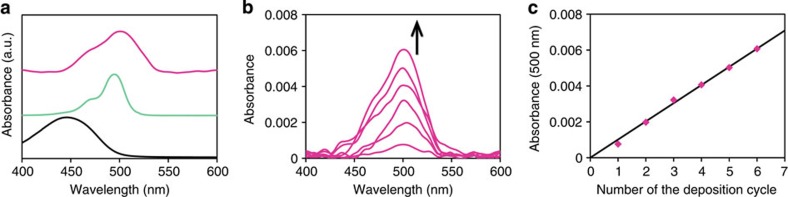
Ultraviolet/visible spectroscopy. (**a**) Spectra of **L1** (black) and **M1** (turquoise) in toluene and few-layer **N1** (magenta) on a quartz substrate. (**b**) Spectral change on stepwise depositions of single-layer **N1** on a quartz substrate. (**c**) Linear relationship between the absorbance at 500 nm and the number of deposition processes. The magenta dots are extracted from Fig. 6b, and the black solid line corresponds to the least-squares linear fit of the plots.

Figure 6a presents the ultraviolet/visible spectra of **L1** and **M1** in toluene and of few-layer **N1** on a quartz substrate. Ligand **L1** displayed an intense absorption band at 446 nm, which is derived from the ^1^π–π* transition of the dipyrrin π-system. Complexation with a zinc(II) ion is known to induce a redshift in the ^1^π–π* band^53^, and in fact **M1** displayed a 49-nm wavelength shift relative to **L1**. Few-layer **N1** also showed an absorption band in the visible region, with the absorption maximum being closer to that of **M1** than to that of **L1**, which also indicates that the complexation of **L1** with zinc(II) ions was complete. Using the ^1^π–π* band as a probe, single-layer **N1** was accumulated stepwise on a quartz substrate. The single-layer nanosheet was fabricated on the air/water interface of a Langmuir–Blodgett trough, which was deposited repeatedly on a quartz substrate at a constant surface pressure using the Langmuir–Schäfer method. Figure 6b presents the ultraviolet/visible spectra of the modified quartz substrate. The peak absorbance of the ^1^π–π* band at 500 nm is proportional to the number of deposition processes (Fig. 6c), which indicates the quantitative, layer-by-layer accumulation of single-layer **N1**.

### Guest inclusion

Uptake of a fluorescent dye, Rhodamine B, to **N1** with large pores was demonstrated (Supplementary Fig. 9). On immersing multi-layer **N1** on an ITO or quartz substrate in a dichloromethane solution of Rhodamine B, **N1** was stained the colour of the dye (Supplementary Fig. 9a). Guest-incorporated **N1** displayed fluorescence from Rhodamine B (Supplementary Fig. 9b,c).

### Photoelectric conversion

**Figure 7:**
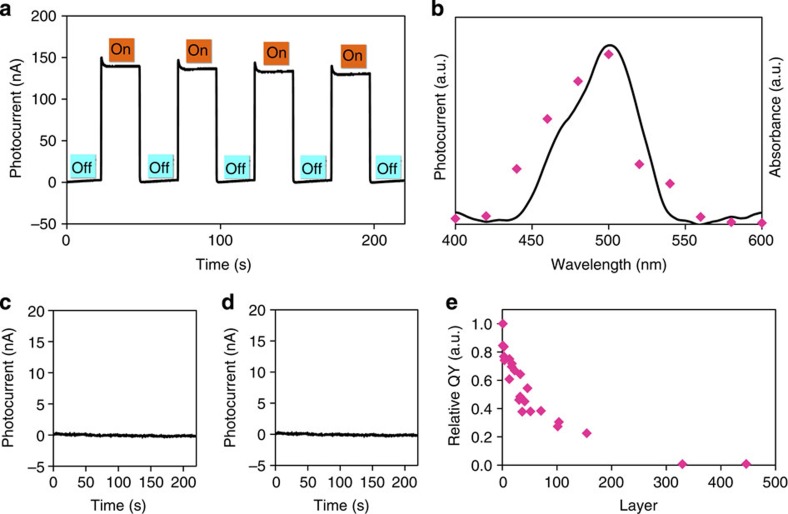
Photoelectric conversion ability of N1. (**a**) Typical anodic current response on irradiation of a working electrode (SnO_2_ substrate modified with 36-layer **N1**) with intermittent 500-nm light. (**b**) Action spectrum for the photocurrent generation (magenta dots) and absorption spectrum of **N1** (black solid line). (**c**) Control experiment without **N1**. (**d**) Control experiment without TEOA. The irradiation pattern is the same as that of **a**. (**e**) Relationship between the relative quantum yield (QY) and thickness of **N1** on irradiation with 500-nm light. The highest QY (0.86% by single-layer **N1**) is taken as the standard.

**N1** was employed as the active layer of a photoanode to demonstrate its functionality. A transparent SnO_2_ working electrode was decorated with 36-layer **N1**, and a three-electrode system was set up (Supplementary Fig. 10). Triethanolamine (TEOA) was added to an electrolyte solution as a sacrificial electron donor. An anodic current was observed only when the working electrode was irradiated with 500-nm light, corresponding to the absorption maximum of **N1** (Fig. 7a). The action spectrum shown in Fig. 7b demonstrates that the photocurrent was maximized with 500-nm light, and no response was observed using light at *λ*<420 nm or *λ*>560 nm, a region of negligible absorption for **N1**. Control experiments lacking either **N1** or TEOA did not show current responses at all (Fig. 7c,d). Therefore, the observed photocurrent is derived from the photocatalytic oxidation of TEOA sensitized by **N1**. Surprisingly, to the best of our knowledge, this report is the first on photoelectric conversion using a bis(dipyrrinato)zinc(II) complex sensitizer despite its excellent light absorption ability. We then studied the relationship between the quantum yield of the photoelectric conversion and thickness of **N1** (Fig. 7e; Supplementary Fig. 11). Single-layer **N1** exhibited the highest value (0.86%), which decreases gradually with the growth of the thickness of **N1**, leading to negligible photoresponses at thicknesses of over ~300 layers. For the photocurrent, the maximum is located at ~100–150 layers (Supplementary Fig. 12). To demonstrate the superiority of **N1**, we also prepared the two types of mononuclear bis(dipyrrinato)zinc(II) complex sensitizers: plain zinc(II) complex **M2**, which was dropcasted onto a SnO_2_ electrode to form a physisorbed film (Supplementary Fig. 13a) and **M3** with carboxy groups, which underwent chemisorption onto a SnO_2_ surface to form a self-assembled monolayer (Supplementary Fig. 14a). These two photoanodes resulted in much lower conversion efficiencies (0.030 and 0.069%, Supplementary Figs 13 and 14), thereby justifying the superiority of bottom-up nanosheet **N1** over conventional molecular films. The nanosheet structure of **N1** affords appropriate porosity and suppresses molecular aggregation; these features presumably make **N1** a better sensitizer. In addition, the coexistence of insolubility (to avoid redissolution into media) and manipulability (to facilitate deposition and layering) of **N1** is also advantageous for potential applications.

## Discussion

A photofunctional bottom-up nanosheet containing the photoactive bis(dipyrrinato)zinc(II) complex motif was fabricated. A nanosheet with atomic thickness was prepared via an air/water interfacial synthesis, during which spontaneous complexation proceeded between a three-way dipyrrin ligand and zinc(II) ions at the interface. The nanosheet was identified using ultraviolet/visible spectroscopy and XPS, which revealed the complete formation of the bis(dipyrrinato)zinc(II) complex motif. The single-layer nanosheet was confirmed by AFM via a scratch experiment. The domain size of the single-layer nanosheet reached 10 μm on one side, which is large for bottom-up nanosheet materials. Repeated deposition of the single-layer nanosheet on a flat substrate resulted in its quantitative layering. The nanosheet efficiently collectes visible light at around 500 nm, and that physisorbed on a transparent SnO_2_ functioned as an active layer in a photoelectric conversion system. The photo-functionality in the molecule-based bottom-up nanosheet demonstrated herein leads to a significant expansion of the applicability of this type of two-dimensional matters as useful and promising nanomaterials.

## Methods

### Materials

5′-(4-Formylphenyl)-[1,1′:3′,1′′-terphenyl]-4,4′′-dicarbaldehyde^56^, 2-methylpyrrole^57^, 2-((3,5-dimethyl-2*H*-pyrrol-2-ylidene)(2,6-dimethylphenyl)methyl)-3,5-dimethyl-1*H*-pyrrole^54^ and 2-methyl-5-((5-methyl-2*H*-pyrrol-2-ylidene)(phenyl)methyl)-1*H*-pyrrole^58^ were synthesized according to previous reports. Dichloromethane, acetonitrile and ethanol for the interfacial synthesis of **N1**, pretreatments of substrates and photoelectric conversion were supplied from Kanto Chemical Co., Inc. as high-performance liquid chromatography grade and were used as received. Water was purified using a Milli-Q purification system (Merck KGaA). Tetra-*n*-butylammonium perchlorate as a supporting electrolyte was purified by recrystallization from ethanol, which was dried in vacuo. Solvents for organic syntheses were purified using a solvent purification system (Ultimate Solvent System, Nikko Hansen & Co., Ltd). The other chemicals were general grades and were used as received. All procedures were conducted under an ambient condition otherwise stated.

### Apparatus for the identification of molecular compounds

^1^H (500 or 400 MHz) and ^13^C (125 or 100 MHz) nuclear magnetic resonance (NMR) spectra were recorded on a Bruker-DRX500, JEOL ECX-400 or JEOL AL-400 spectrometer. Fast atom bombardment mass spectrometry (FAB-MS) and electrospray ionization time-of-flight spectrometry were conducted using a JEOL JMS-700 MStation and Micromass LCT Premier XE mass spectrometer, respectively.

### Synthesis of L1

5′-(4-Formylphenyl)-[1,1′:3′,1′′-terphenyl]-4,4′′-dicarbaldehyde (450 mg, 1.2 mmol) and 2-methylpyrrole (0.63 ml, 7.5 mmol) were dissolved in dichloromethane (100 ml) under a nitrogen atmosphere. One drop of trifluoroacetic acid was added, and the solution changed from light yellow to bright red and was stirred at room temperature for 3 h. When complete consumption of the aldehyde was confirmed by thin-layer chromatography, a solution of chloranil (848 mg, 3.4 mmol) in dichloromethane was added, and the resultant mixture was stirred for an additional 15 min. The reaction mixture was washed with water, dried over magnesium sulfate, filtered and evaporated. The crude product was purified by column chromatography on aluminum oxide (activity II–III) with dichloromethane as an eluent to yield a deep-yellow powder (231 mg, 25%). ^1^H NMR (400 MHz, CDCl_3_): *δ* 7.95 (s, 3H), 7.80 (d, *J*=8.0 Hz, 6H), 7.61 (d, *J*=8.0 Hz, 6H), 6.56 (d, *J*=4.0 Hz, 6H), 6.19 (d, *J*=4.0 Hz, 6H), 2.47 (s, 18H); ^13^C NMR (100 MHz, CDCl_3_): *δ* 165.18, 146.78, 141.99, 137.88, 135.32, 132.12, 130.51, 128.89, 127.12, 116.54, 112.11, 105.97 and 18.21; HR-FAB-MS (*m*/*z*): [M]^+^ calculated for C_57_H_48_N_6_, 816.3940; found, 816.3924.

### Synthesis of multi-layer N1

A 50-ml cylindrical glass vial (3.2 cm in diameter) was used as the reaction container. **L1** was dissolved in dichloromethane to a concentration of 1.0 × 10^−4^ mol l^−1^, and 10 ml of the solution was poured into the vial. Then, pure water (10 ml) was layered gently onto the organic phase, which served as a buffer layer. After 2 h, aqueous zinc(II) acetate solution (0.1 mol l^−1^, 10 ml) was slowly added to the buffer layer. The reaction system was left undisturbed for 4 days to obtain an orange film at the liquid/liquid interface. The resulting multi-layer **N1** could be deposited on various substrates.

### Single-phase reactions between L1 and zinc(II) acetate

Two types of reaction were performed. Stoichiometric amounts of zinc(II) acetate dihydrate (4.9 mg, 0.022 mmol) and **L1** (12.3 mg, 0.015 mmol) were dissolved in dichloromethane (5 ml) in a glass vial. The reaction mixture was allowed to stand at room temperature for 1 day. The resultant product was filtered and washed with dichloromethane, ethanol and water to yield a brown solid (Fig. 2f). In the other type of reaction, stoichiometric amounts of zinc(II) acetate dihydrate (5.1 mg, 0.023 mmol) and **L1** (12.2 mg, 0.015 mmol) were dissolved in *N*,*N*-dimethylformamide (5 ml) in a glass vial. After sealing the vial tightly, the reaction mixture was heated at 105 °C for 1 day in an oil bath. After being cooled down to room temperature, the resultant product was filtered and washed with dichloromethane, *N*,*N*-dimethylformamide and water to obtain a brown solid (Fig. 2g).

### Synthesis of single-layer or few-layer N1

A 50-ml glass vial (3.2 cm in diameter) was used as the reaction container. Aqueous zinc(II) acetate (0.05 mol l^−1^, 30 ml) was poured into the vial. A small amount of a dichloromethane solution of **L1** was then sprinkled gently on the surface of the aqueous phase: single-layer **N1** was synthesized using 5.0 μl of **L1** solution (7.4 × 10^−5^ mol l^−1^), whereas 20 μl of **L1** solution (7.4 × 10^−5^ mol l^−1^) was used for the few-layer nanosheet. After spontaneous evaporation of the organic solvent, the reaction system was left undisturbed, such that **N1** was produced at the air/liquid interface. Single-layer or few-layer **N1** was then transferred onto substrates using the Langmuir–Schäfer method.

### Synthesis of mononuclear bis(dipyrrinato)zinc(II) complex M1

Zinc(II) acetate dihydrate (36.7 mg, 0.17 mol) and triethylamine (0.11 ml) were added to a dichloromethane solution (20 ml) of 2-((3,5-dimethyl-2*H*-pyrrol-2-ylidene)(2,6-dimethylphenyl)methyl)-3,5-dimethyl-1*H*-pyrrole (122 mg, 0.40 mmol), and the reaction mixture was stirred overnight at room temperature. Methanol (20 ml) was added to recrystallize the product as an orange solid (60.5 mg, 45%). ^1^H NMR (500 MHz, CDCl_3_): *δ* 7.21 (t, *J*=7.6 Hz, 2H), 7.11 (d, *J*=7.6 Hz, 4H), 5.91 (s, 4H), 2.16 (s, 12H), 2.04 (s, 12H) and 1.28 (s, 12H); ^13^C NMR (125 MHz, CDCl_3_): *δ* 156.07, 143.26, 143.06, 139.18, 135.95, 134.27, 127.98, 127.88, 119.74, 19.33, 16.13 and 14.70; HR-FAB-MS (*m*/*z*): [M]^+^ calculated for C_42_H_46_N_4_Zn, 670.3014; found, 670.3011.

### Synthesis of mononuclear bis(dipyrrinato)zinc(II) complex M2

A solution of 2-methyl-5-((5-methyl-2*H*-pyrrol-2-ylidene)(phenyl)methyl)-1*H*-pyrrole (450 mg, 1.8 mmol) in methanol (10 ml) was poured into a suspension of anhydrous zinc(II) acetate (168 mg, 0.92 mmol) in methanol (3 ml). The mixture was stirred overnight at room temperature. The reaction mixture was filtered, and the resultant solid was washed with methanol. Filtration yielded a light orange solid (463 mg, 90%). ^1^H NMR (400 MHz, CDCl_3_): *δ* 7.50−7.39 (m, 10H), 6.55 (d, *J*=4.0 Hz, 2H), 6.18 (d, *J*=4.0 Hz, 2H) and 2.12 (s, 12H); ^13^C NMR (100 MHz, CDCl_3_): *δ* 159.44, 144.71, 139.52, 139.39, 133.22, 130.89, 128.17, 127.14, 117.10 and 16.62; HR-FAB-MS (*m*/*z*): [M]^+^ calculated for C_42_H_46_N_4_Zn, 558.1762; found, 558.1782.

### Synthesis of a precursor for M3

To a dichloromethane (20 ml) solution of ethyl 3-(4-formylphenyl)propanoate (618 mg, 3.0 mmol) and 2-methylpyrrole (0.55 ml, 6.3 mmol), trifluoroacetic acid (10 μl) was added, under a nitrogen atmosphere, and the solution was stirred overnight at room temperature. Chloranil (749 mg, 3.0 mmol) was added and stirred for another 30 min. After evaporation of the solvent, the residue was passed through a short pad of aluminum oxide (activity II–III) using dichloromethane as an eluent. After evaporation of the solvent, the crude product was purified by column chromatography on aluminum oxide (activity II–III) with hexane/dichloromethane (1:1 v/v) as an eluent. The yellow band was collected and evaporated to yield ethyl 3-(4-((5-methyl-1H-pyrrol-2-yl)(5-methyl-2H-pyrrol-2-ylidene)methyl)phenyl)propanoate as an orange oil (146 mg, 14%). ^1^H NMR (500 MHz, CDCl_3_) *δ* 7.41 (d, *J*=8.2 Hz, 2H), 7.26 (d, *J*=8.2 Hz, 2H), 6.48 (d, *J*=3.8 Hz, 2H), 6.17 (d, *J*=3.8 Hz, 2H), 4.18 (q, *J*=7.3 Hz, 2H), 3.05 (t, *J*=7.9 Hz, 2H), 2.71 (t, *J*=7.9 Hz, 2H), 2.46 (s, 6H) and 1.28 (t, *J*=7.3 Hz, 3H); ^13^C NMR (125 MHz, CDCl_3_) *δ* 172.84, 153.67, 140.99, 139.91, 138.30, 135.49, 130.99, 128.96, 127.41, 117.35, 60.49, 35.81, 30.79, 16.29 and 14.27; HR-FAB-MS (*m*/*z*): [M]^+^ calculated for C_22_H_24_N_2_O_2_, 348.1829; found, 348.1838.

### Synthesis of M3

Ethyl 3-(4-((5-methyl-1*H*-pyrrol-2-yl)(5-methyl-2*H*-pyrrol-2-ylidene)methyl)phenyl)propanoate (100 mg, 0.29 mmol) was added to tetrahydrofuran (30 ml) and water (5 ml), and the mixture was stirred at room temperature for 5 min. Then, an aqueous solution (10 ml) of NaOH (115 mg, 2.9 mmol) was added, and the reaction mixture was stirred overnight at room temperature. After adjusting the pH of the reaction mixture to 4 using aqueous hydrochloric acid (1.0 × 10^−4^ mol l^−1^), the mixture was poured into water (50 ml) and washed with ether (20 ml × 3). The water layer was then treated with a solution of anhydrous zinc(II) acetate (58 mg, 0.29 mmol) in water (3 ml). The mixture was extracted with dichloromethane and dried over sodium sulfate. The solvent was evaporated under a reduced pressure to yield a dark orange solid of **M3** (3.0 mg, 3%). ^1^H NMR (500 MHz, dimethylsulfoxide-d_6_): *δ* 12.34 (bs, 2H), 7.34–7.30 (m, 8H), 6.33 (d, *J*=4.0 Hz, 4H), 6.20 (d, *J*=4.0 Hz, 4H), 2.91 (t, *J*=7.6 Hz, 4H), 2.61 (t, *J*=7.6 Hz, 4H) and 2.39 (s, 12H); ^13^C NMR (125 MHz, CDCl_3_) *δ* 158.99, 144.79, 139.06, 133.29, 130.90, 130.59, 127.60, 117.85, 31.16, 25.62 and 16.49 (two quaternary carbons are missing because of low solubility or overlaps with other peaks); HR-ESI-MS (*m*/*z*): [M-H^+^] calculated for C_40_H_37_N_4_O_4_Zn, 701.2106; found, 701.2122.

### Pretreatments for substrates

HOPG was cleaved with a piece of cellophane tape to obtain a flat and clean surface just before use. Si(111) substrates were hydrophobized using HMDS. The procedure to form a HMDS/Si(111) surface is as follows. The Si(111) substrate covered with a natural SiO_2_ layer was sonicated in acetone (10 min), 2-propanol (15 min × 2), water (15 min) and ethanol (10 min). After rinsing the substrate in ethanol a couple of times, the substrate was immersed in an ethanol solution of HMDS (1 v/v%) for 1 h. The substrate was then washed with ethanol several times before being annealed at 130 °C for 30 min. The modified substrate was then sonicated in ethanol (15 min) and water (10 min) and was stored in water. Just before use, HMDS/Si(111) was sonicated in 2-propanol (15 min) and water (15 min), which was dried in vacuo overnight. ITO (on glass), quartz and SnO_2_ (on ITO-covered glass, 5 Ω sq^−1^) substrates were sonicated in acetone (10 min × 2), water (10 min × 2) and nonionic detergent in water (30 min). Then, the substrate was washed with water until the bubbles of the detergent disappeared before being sonicated in water (10 min × 3) and ethanol (10 min × 2). The cleaned substrate was stored in water, and dried by nitrogen blow just before use.

### Layering of single-layer N1

A Langmuir–Blodgett trough (KSV 2000, KSV NIMA) was used as a reaction container. Before a zinc(II) acetate aqueous solution (0.05 mol l^−1^) was poured into the trough as a subphase, the trough was washed with ethanol. The surface of the aqueous solution was cleaned by suctioning the surface of the subphase several times on compressing the trough barrier. Then, a dichloromethane solution of **L1** (7.4 × 10^−5^ mol l^−1^, 27.7 μl) was dropped gently onto the surface of the subphase. Under this condition, the ideal coverage of single-layer **N1** reached 90% of the trough area. After spontaneous evaporation of the organic solvent, the reaction system was left undisturbed for 4 h, such that single-layer **N1** was produced at the air/liquid interface. The trough was then compressed to yield a trough area of 69.8 cm^2^ (90% of the initial trough area) at a surface pressure of 0.04 mN m^−1^. Under these conditions, single-layer **N1** was transferred onto a quartz substrate via the Langmuir–Schäfer method. The trough was then shrunk by the same area as the substrate, and the next transfer was conducted. This process was repeated to layer single-layer **N1**.

### Guest inclusion

Multi-layer **N1** (thickness: 700 nm) was deposited on a quartz or ITO substrate. The modified substrate was immersed in a dichloromethane solution of Rhodamine B (5.0 × 10^−5^ mol l^−1^) for 15 h and was then rinsed with dichloromethane to yield Rhodamine B-encapsulated multi-layer **N1**.

### Analyses for N1

XPS was conducted using a PHI 5,000 VersaProbe (Ulvac-Phi, Inc.). Al Kα (15 kV, 25 W) was used as the X-ray source, and the beam was focused on a 100-μm^2^ area. The spectra were analysed with MultiPak Software and standardized using the C 1*s* peak at 284.6 eV. AFM and STM were performed using an Agilent Technologies 5,500 scanning probe microscope under an ambient condition. AFM was performed in high-amplitude mode (tapping mode) with a silicon cantilever PPP-NCL (Nano World). The probe for STM (Pt-Ir alloy, 4:1, 0.25 mm in diameter) was cut from a wire using a nipper to obtain a sharp edge. An optical microscope image was taken using a VHX-100 (Keyence Corporation). A field-emission scanning electron microscopic image was collected using a JEOL JSM-7400FNT. Transmission electron microscopy images/SAED patterns were recorded at accelerating voltage of 75 kV using a Hitachi HF-2000 equipped with an AMT-CCD camera. The sample was prepared by depositing multi-layer **N1** (thickness: 700 nm) on a carbon film supported by a copper grid (ELS-C10, stem Co., Ltd) directly from the liquid/liquid interface. To acquire electron diffractions, we focused on the edge of multi-layer **N1**. To reproduce the obtained SAED pattern, three-dimensional structures of multi-layer **N1**, which comprise piles of single-layer **N1**, were considered (given as a Supplementary Information and shown in Supplementary Fig. 8). Here we treated AA-, AB- and ABC-stack models, which are often encountered in layered materials^59^,^60^. The three-dimensional lattice was optimized at the molecular mechanics level of theory with the UFF VALBOND 1.1 force field on an Accelrys Cerius2 ver3.1 program package. The unit cell was assumed to be trigonal such that the *α*, *β*, and *γ* angles were constrained to be 90°, 90°, and 120°, respectively. First, we performed a calculation on the AA-stacking structure with its molecular geometries and cell lengths being fully optimized. Initial structures for the AB- and ABC-stack models were constructed from the optimized AA-stack one by giving parallel displacement to the B and/or C layers. The SAED patterns were simulated by implementing CrystalMaker 2.6.3 and SingleCrystal 2.3 (CrystalMaker Software Ltd) (Supplementary Fig. 7b–d). Ultraviolet/visible absorption spectra were recorded on a JASCO V-570 spectrometer in transmission mode. A quartz or SnO_2_ substrate modified with **N1** was set vertical to the probe light. Fluorescence and excitation spectra were recorded on a HITACHI F-4500 fluorospectrometer.

### Photoelectric conversion

**N1** with thicknesses ≤155 layers was deposited on a transparent SnO_2_ electrode using the repeated Langmuir–Schäfer procedure shown in Fig. 6b,c. On the other hand, **N1** with thicknesses of >155 layers was fabricated directly using the liquid/liquid interfacial synthesis (Fig. 2a) and deposited on the electrode. Before photoelectric conversion, each **N1** physisorbed on a transparent SnO_2_ electrode was always subjected to ultraviolet/visible spectroscopy, acquiring spectra from four different positions. This series of measurements allowed us to ensure the uniformity of **N1** and to quantify the thickness of **N1** using the average absorbance at 500 nm and that of single-layer **N1** (0.00101 from the slope of Fig. 6c). The average absorbance at 500 nm was also used in calculating the quantum yield of photoelectric conversion (*vide infra*). The modified SnO_2_ electrode was used as a working electrode (photoanode). Silver and platinum wires were employed as reference and counter electrodes, respectively. The resulting three-electrode system was immersed in a homemade photoelectrochemical cell (Supplementary Fig. 10), which was filled with an acetonitrile solution of tetra-*n*-butylammonium perchlorate (0.1 mol l^−1^) containing TEOA (0.05 mol l^−1^) as a sacrificial donor reagent. The cell was sealed and deoxygenized by argon bubbling for 30 min. Monochromatic light for the action spectrum shown in Fig. 7b (400–600 nm in every 20 nm) was extracted from a xenon lamp (MAX–302, Asahi Spectra Co., Ltd), the photon flux of which was monochromated by a monochromator (CT-10, JASCO Corporation). For the other experiments, 500-nm light was used exclusively, which was provided by the xenon lamp equipped with a band-pass filter. The active area of the electrode was 0.264 cm^2^, which was determined by a fluorocarbon rubber o-ring (Supplementary Fig. 10). The electrode potential and photocurrent acquisition of the photoelectric conversion system were controlled using an electrochemical analyser (ALS 750A, BAS Inc.). The photoanode was fixed at 0.0 V versus the silver reference electrode for the data shown in Fig. 7a–d and 0.15 V for the other data, including those for **M2** and **M3**. At both potentials, no anodic current induced by the direct oxidation of TEOA in the dark was observed. The quantum yield of photoelectric conversion, *φ*, was calculated using equation (1):


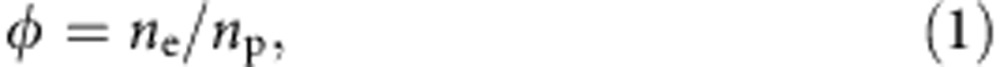


where *n*_e_ is the mole of electrons that flows in the circuit per unit time (in mol s^−1^) and *n*_p_ is the mole of photons absorbed by the sensitizer per unit time (in mol s^−1^). *n*_e_ and *n*_p_ were calculated using equations (2) and (3):


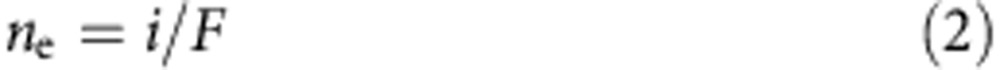



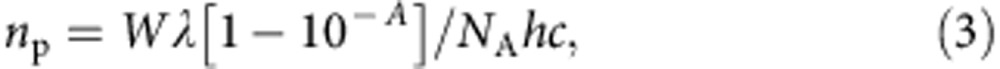


where *i* is the current flow (in A), *F* is the Faraday constant (9.65 × 10^4^ C mol^−1^), *W* is the photon flux of incident light (in J s^−1^), *λ* is the wavelength of the irradiated light (5.00 × 10^−7^ m), *A* is the absorbance at the irradiated wavelength, *N*_A_ is the Avogadro constant (6.02 × 10^23^ mol^−1^), *h* is the Planck constant (6.63 × 10^−34^ Js) and *c* is the velocity of light (3.00 × 10^8^ m s^−1^). A representative data set for the determination of *φ* for **N1** is shown in Supplementary Fig. 11. *i* was calculated using equation (4):


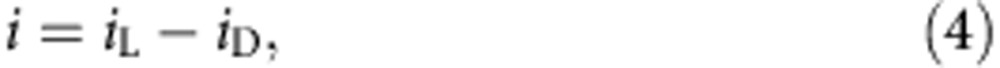


where *i*_L_ is the average light current for the first cycle (10 s) and *i*_D_ is the average dark current just before the illumination of light. A photon counter (8230E and 82311B, ADC Corporation) was employed for the quantification of *W*. For every sample, *W* was measured independently. A typical value for *W* was ~0.20 mW. When *i* was below the measurable level (<1.0 nA), *W* was increased to 1.0–2.0 mW such that the photocurrent signal was amplified. For referential mononuclear complex **M2**, film formation was conducted as follows: two droplets from a disposable Pasteur pipette of an acetonitrile solution of **M2** (1.86 × 10^−5^ mol l^−1^) were dripped onto a SnO_2_ substrate (~0.8 cm^2^). The substrate was then dried under vacuum for 10 min to allow the solvent to evaporate. The modified SnO_2_ electrode was subjected to photoelectric conversion, following the procedure for **N1** except that aqueous 0.1 mol l^−1^ sodium sulfate was used as an electrolyte solution; this change prevented the **M2** film from redissolution. Referential mononuclear complex **M3** was immobilized on a SnO_2_ electrode through the carboxy group using the self-assembled monolayer procedure^61^. A SnO_2_ substrate was immersed in a dimethylsulfoxide solution of **M3** (1.96 × 10^−3^ mol l^−1^) for 1 day at room temperature, and the decorated substrate was rinsed with dimethylsulfoxide and dried by an argon blow. The modified SnO_2_ electrode was subjected to photoelectric conversion using the same procedure as **N1**.

## Author contributions

R.S. conceived and directed the project. R.S., H.N., K.H. and W.-Y.W. initiated the project. S.K., Q.L. and W.-Y.W. synthesized ligand **L1**. K.H., Q.L. and T.N. performed the fabrication of **N1** and **M1**. T.N. and M.T. synthesized **M2** and **M3**, respectively. Y.K. optimized the three-dimensional lattice of multi-layer **N1** at the molecular mechanics level of theory. K.H. conducted the characterization of **N1**. T.Y. and K.H. investigated the photoelectric conversion of **N1**, **M2** and **M3**. R.S. and K.H. co-wrote the manuscript.

## Additional information

**How to cite this article:** Sakamoto, R. *et al.* A photofunctional bottom-up bis(dipyrrinato)zinc(II) complex nanosheet. *Nat. Commun.* 6:6713 doi: 10.1038/ncomms7713 (2015).

## Supplementary Material

Supplementary Information Supplementary Figures 1-14 and Supplementary References
