# Supplementary material for: A photofunctional bottom-up bis(dipyrrinato)zinc(II) complex nanosheet
Source: Nat Commun. 2015 Apr 2;6:6713. doi: 10.1038/ncomms7713 (PMC4396372; doi:10.1038/ncomms7713)
Supplement: Supplementary Information — Supplementary Figures 1-14 and Supplementary References [file ncomms7713-s1.pdf]

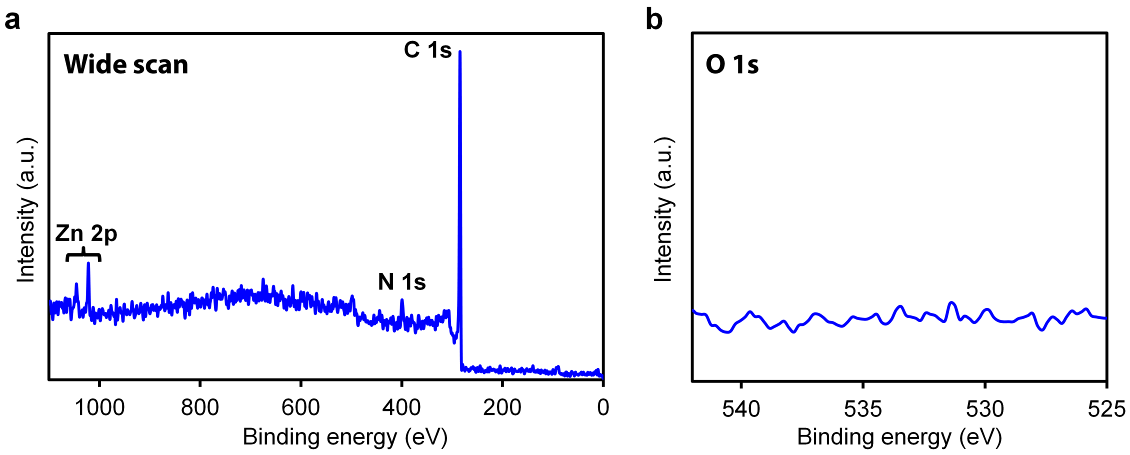

Supplementary Figure 1 | XPS of multi-layer N1 on HOPG. (**a**) Wide-scan. (**b**) Narrow-scan focusing on the O 1s region. The sample was subjected to argon sputtering to remove the outermost layer contaminated by oxygen and/or water. The vertical axis is not corrected using the ionization cross-section.

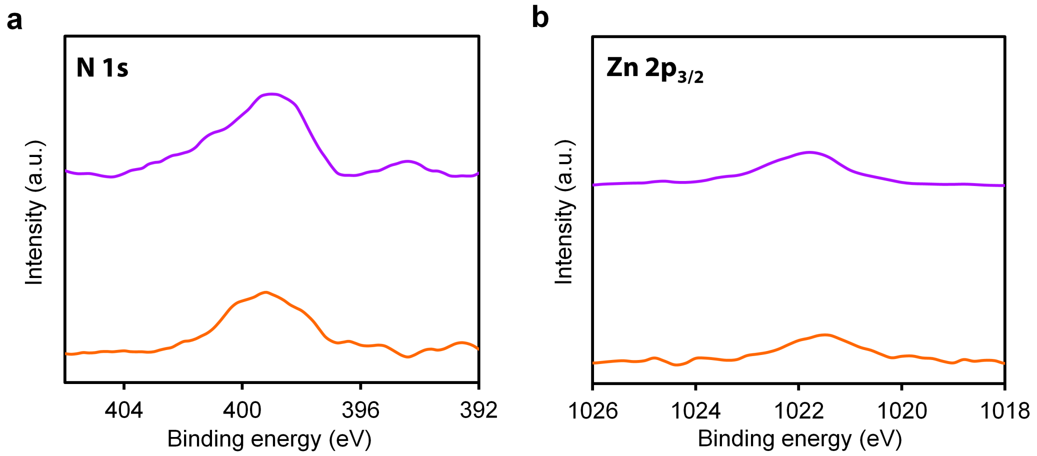

Supplementary Figure 2 | XPS of the products of the one-phase reactions. (**a**) Narrow-scan focusing on the N 1s region. (**b**) Focusing on the Zn $2p_{3/2}$ region. Orange solid line: synthesized at room temperature in dichloromethane (Fig. 2f), purple solid line: 105°C in *N*,*N*-dimethylformamide (Fig. 2g). The sample was immobilized on a piece of conductive adhesive carbon tape. The intensity is proportional to the element abundance: The original signal is standardized using the photoionization cross-section of each element. N : Zn = 5.4 : 1 (84.4 : 15.6) for room temperature in dichloromethane and 5.8 : 1 (85.3 : 14.7) for 105°C in *N*,*N*-dimethylformamide.

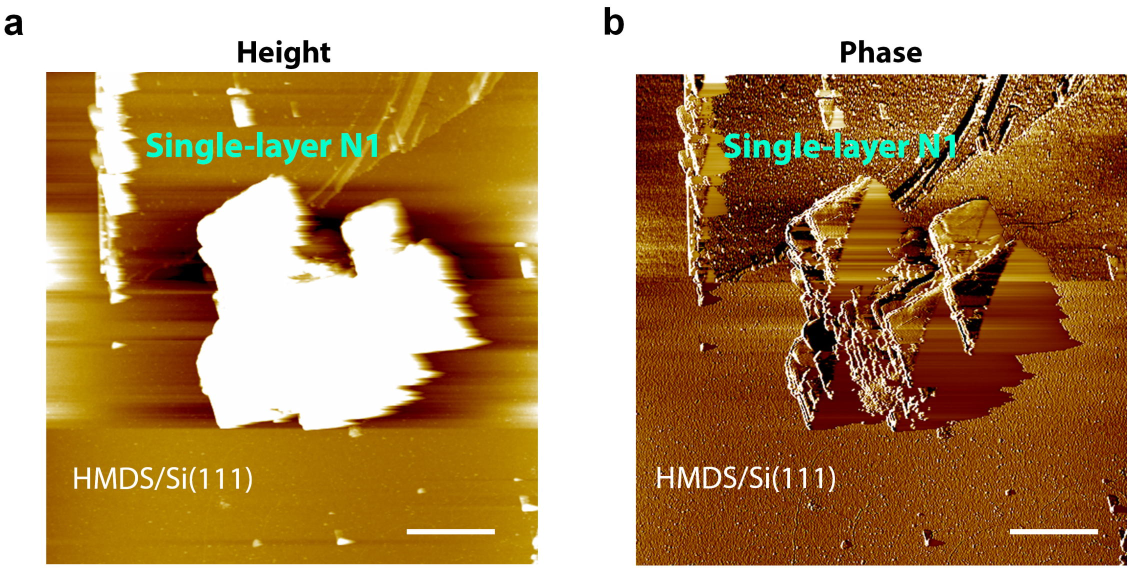

Supplementary Figure 3 | AFM images of N1 on HMDS/Si(111) scratched by an AFM tip at a force of 1.2×10^4 nN. (**a**) Height image. (**b**) Phase image. This scratch ended in the destruction of both **N1** and HMDS/Si(111) surface. Scale bar, 1 μm.

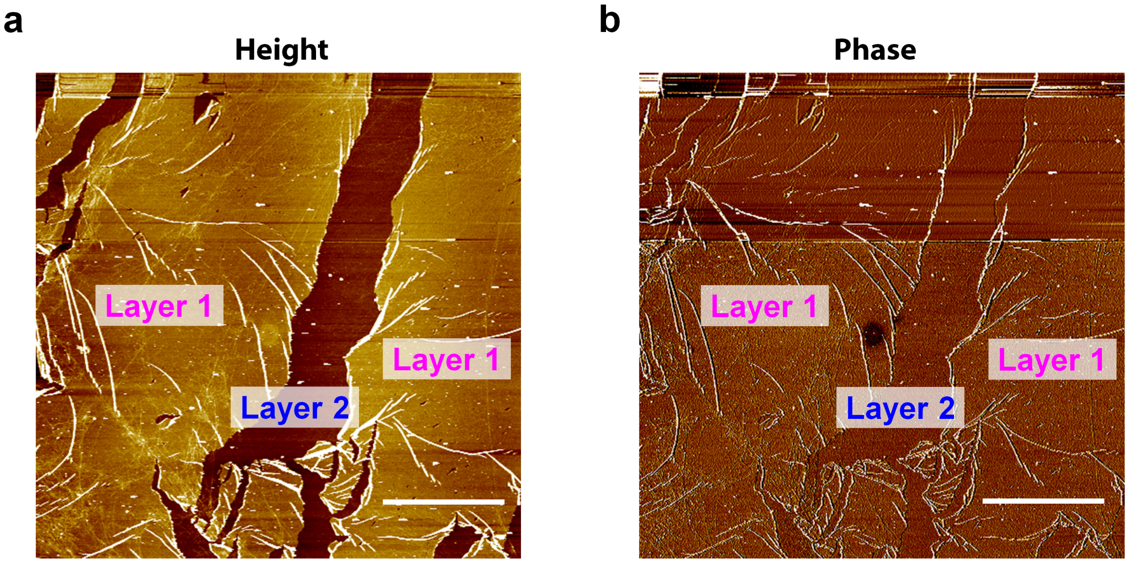

Supplementary Figure 4 | AFM image of few-layer N1 with large domain sizes on HMDS/Si(111). (**a**) Height image. (**b**) Phase image. In the height image, at least two-layers can be recognized (Layers 1 and 2). Because the phase image is flat, both layers are assignable to **N1**, covering the entire surface in the scanned region. Scale bar, 5 μm.

Height

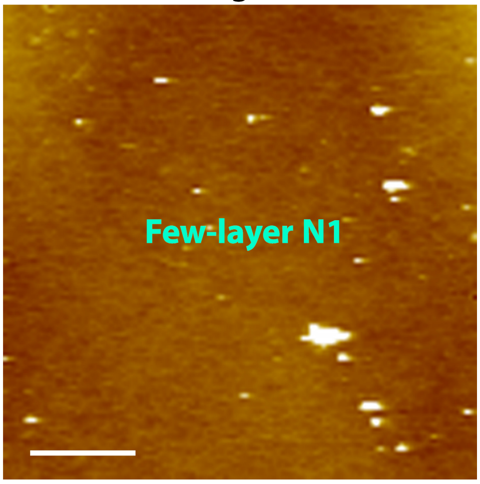

Supplementary Figure 5 | AFM height image of few-layer N1 on HMDS/Si(111) after thermal annealing at 120°C for 3 days. The entire surface of HMDS/Si(111) is covered with **N1**. The wrinkles observed in Supplementary Fig. S4 are removed. Scale bar, 1 µm.

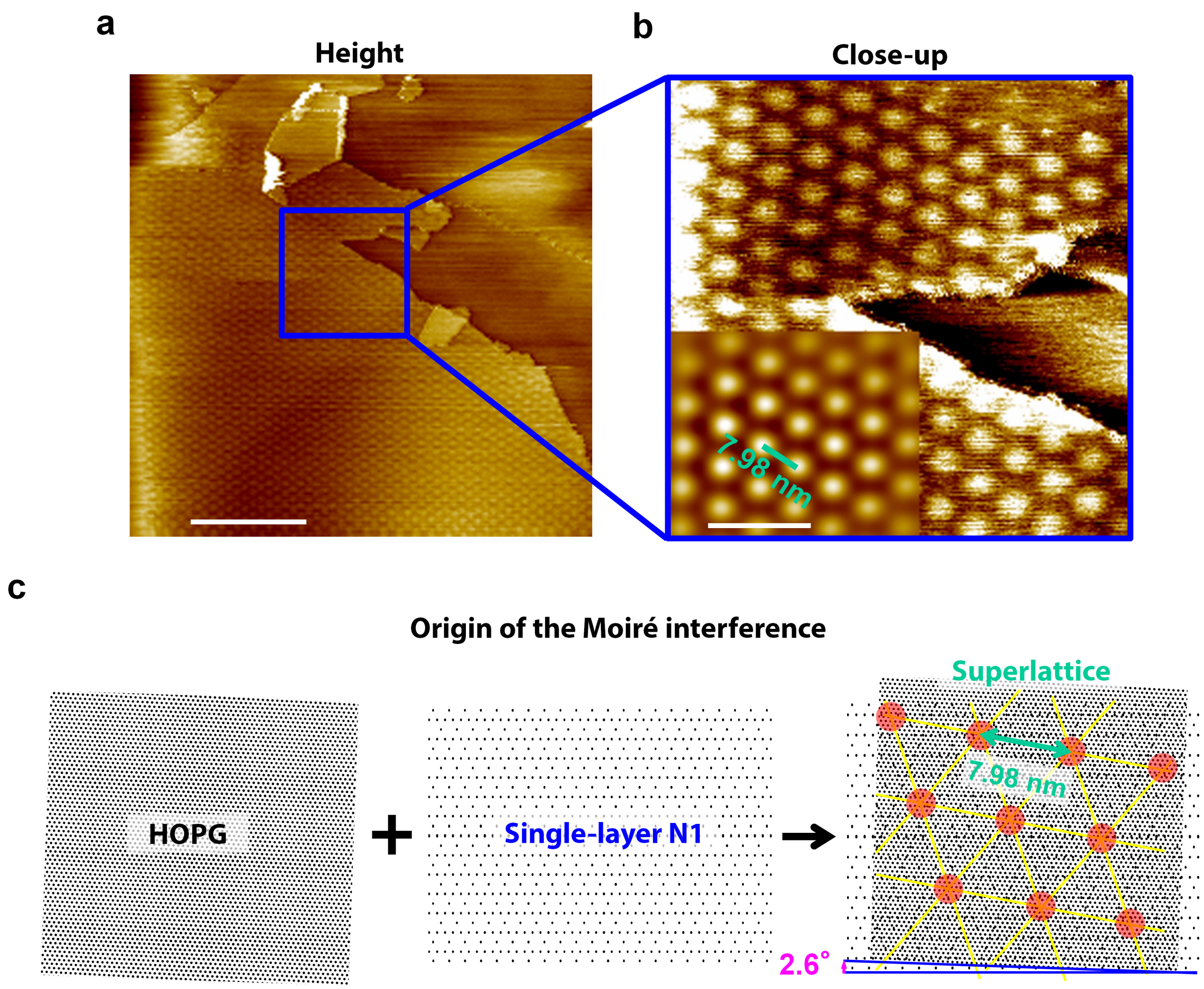

Supplementary Figure 6 | STM image of single-layer N1 on HOPG. (**a**) Height image exhibiting a hexagonal pattern. Scale bar, 100 nm. (**b**) Close-up of the height image. The bottom left corner shows a fast Fourier transform (FFT)-filtered image. Scale bar, 20 nm. The periodicity of the pattern is 7.98 nm, which is too large to assign as the direct structure of single-layer **N1** (3.58 nm, Supplementary Fig. 8). Therefore, the observed hexagonal pattern is derived from moiré interference between two hexagonal lattices, HOPG (0.246 nm) and single-layer **N1**. (**c**) Schematic illustration of the overlap between single-layer **N1** and the HOPG lattice that produces the observed moiré superlattice. The rotation angle is 2.6°. Details of the analysis are provided in previous literature references.[1,2]

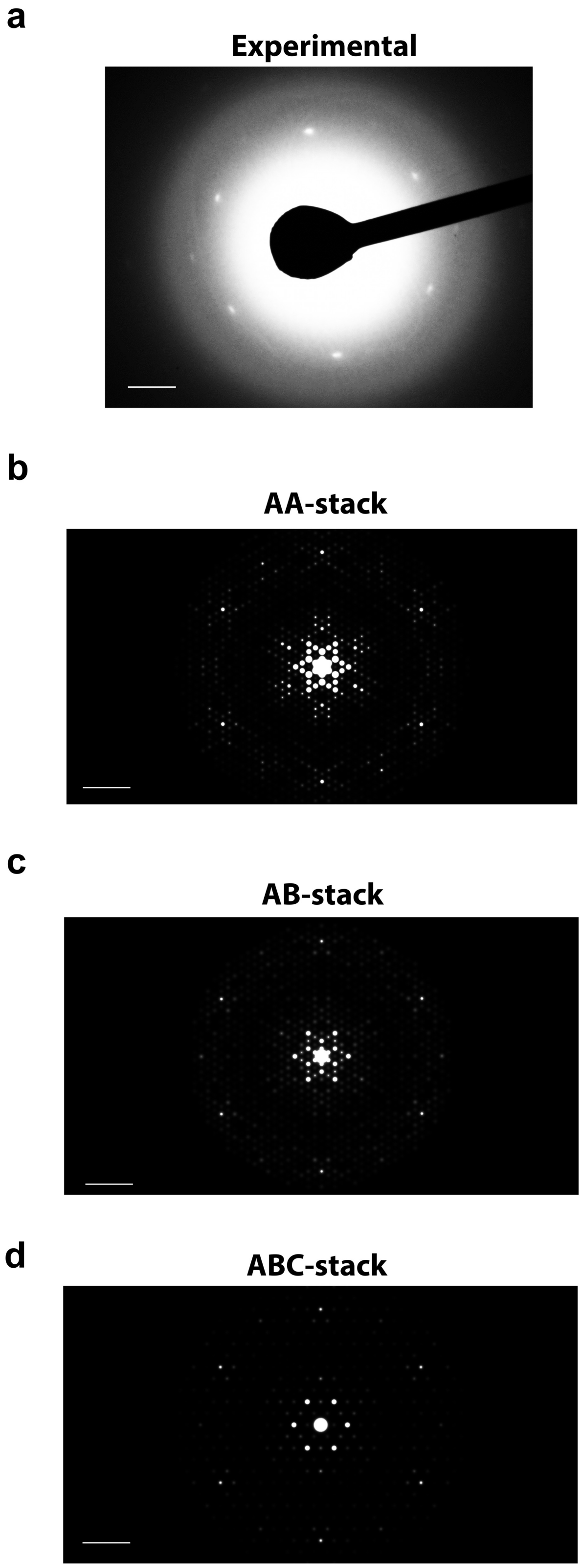

Supplementary Figure 7 | TEM/SAED pattern of multi-layer N1 at room temperature. (**a**) Recorded with a nominal camera length of 20 cm. Lower-index diffractions are disturbed by a direct electron beam. Scale bar, 2 nm^{-1}. (**b-d**) Simulated from the crystal lattices shown in Supplementary Fig. 8. Viewed along the [001] axis. Scale bar, 2 nm^{-1}.

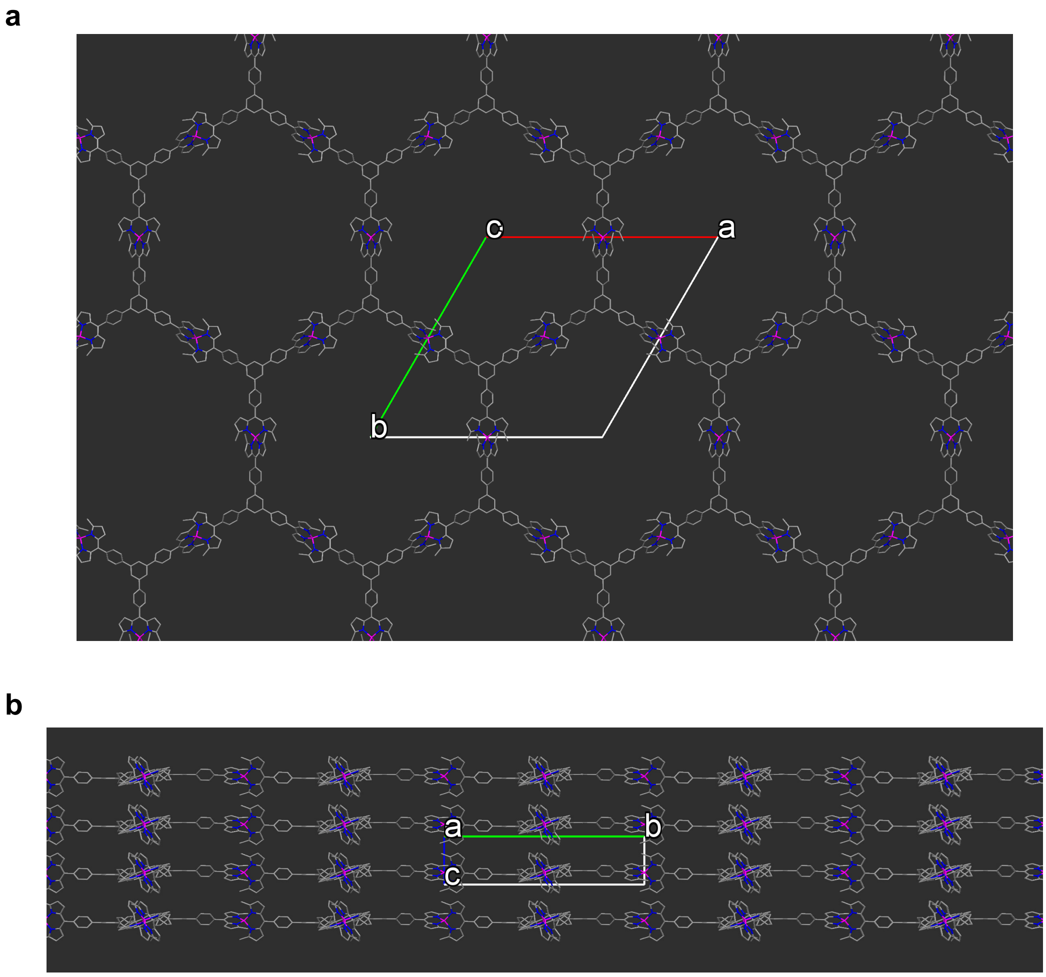

Supplementary Figure 8 | Three-dimensional lattices of N1 optimized using molecular mechanics (UFF VALBOND 1.1). (**a,b**) AA-stack structure viewed along the *c* and *a* axes, respectively. The unit cell takes a pseudo-trigonal symmetry: $a = 35.9$ Å, $b = 35.8$ Å, $c = 7.46$ Å, $\alpha = \beta = 90°$, $\gamma = 120°$. Single-layer **N1** is stacked in the eclipsed fashion. Gray, blue, and magenta correspond to carbon, nitrogen, and zinc, respectively.

c

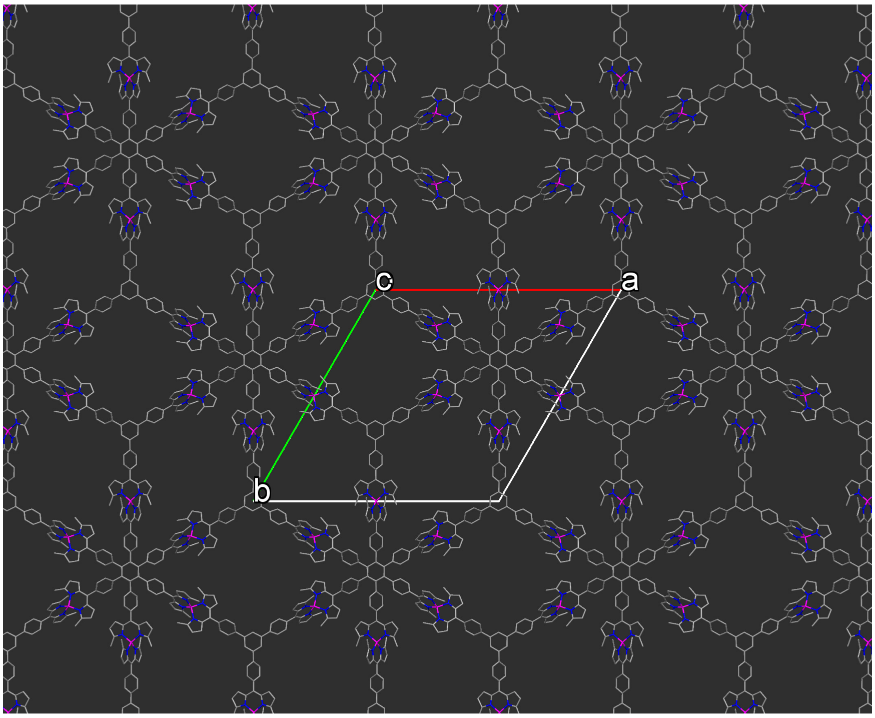

d

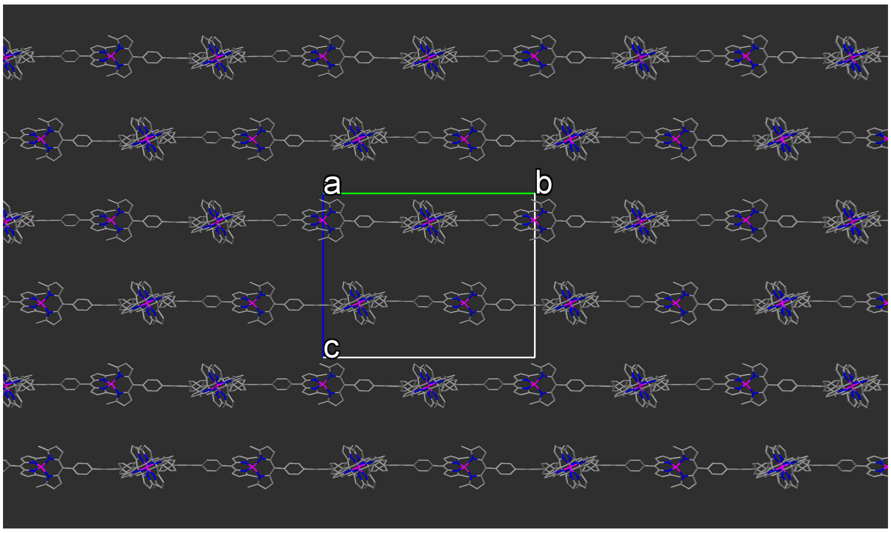

Supplementary Figure 8 | Three-dimensional lattices of N1 optimized using molecular mechanics (UFF VALBOND 1.1) (continued). (**c,d**) AB-stack structure viewed along the *c* and *a* axes, respectively. The unit cell takes a pseudo-trigonal symmetry: $a = 36.0$ Å, $b = 35.8$ Å, $c = 24.0$ Å, $\alpha = \beta = 90°$, $\gamma = 120°$. Single-layer **N1** is stacked in a staggered fashion with a translation vector of 1/2(**a** + **b** + **c**). Gray, blue, and magenta correspond to carbon, nitrogen, and zinc, respectively.

e

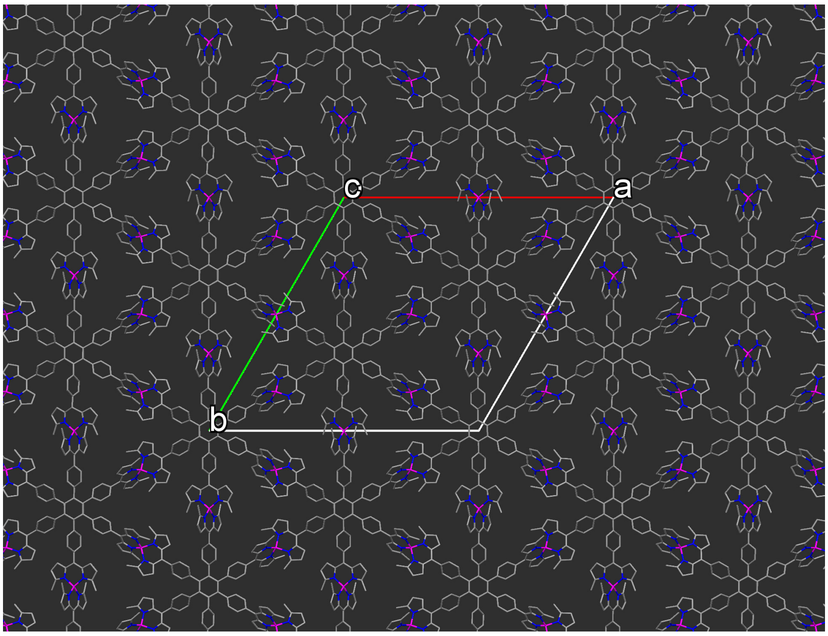

f

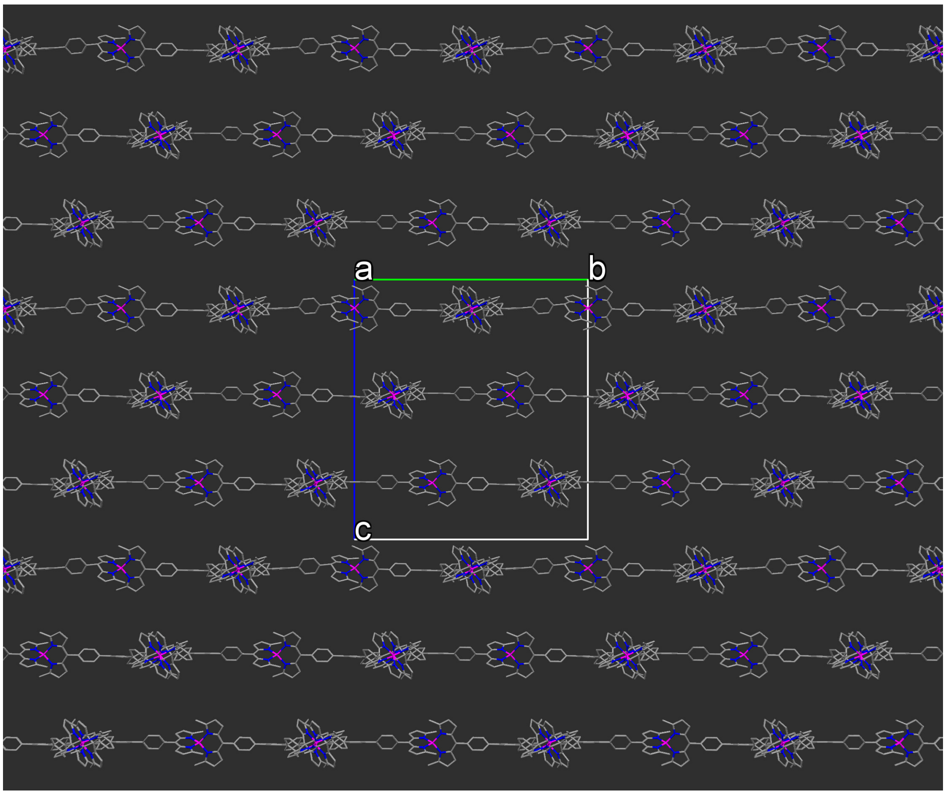

Supplementary Figure 8 | Three-dimensional lattices of N1 optimized using molecular mechanics (UFF VALBOND 1.1) (continued). (**e,f**) ABC-stack structure viewed along the *c* and *a* axes, respectively. The unit cell takes a pseudo-trigonal symmetry: $a = 35.8$ Å, $b = 35.8$ Å, $c = 34.5$ Å, $\alpha = \beta = 90°$, $\gamma = 120°$. Single-layer **N1** is stacked in a staggered fashion with a translation vector of 1/3(**a** + **b** + **c**). Gray, blue, and magenta correspond to carbon, nitrogen, and zinc, respectively.

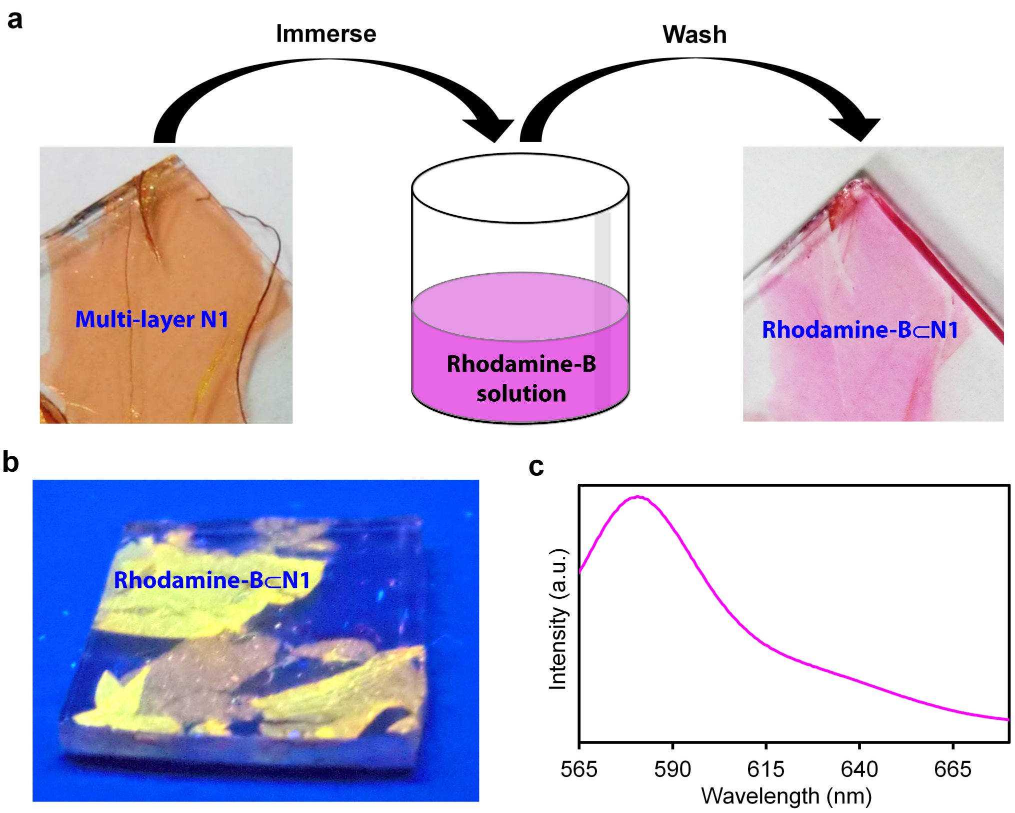

Supplementary Figure 9 | Inclusion of fluorescent guest molecules in multi-layer N1. (**a**) Schematic illustration of the uptake process of Rhodamine B in multi-layer **N1** (**Rhodamine-B⊂N1**) on transparent substrates. (**b**) Photographs of **Rhodamine-B⊂N1** on a quartz substrate under black light with a wavelength of 365 nm. The yellow fluorescence derived from Rhodamine B is conspicuous. (**c**) Fluorescence spectrum of **Rhodamine-B⊂N1** on a quartz substrate upon excitation with 543-nm light.

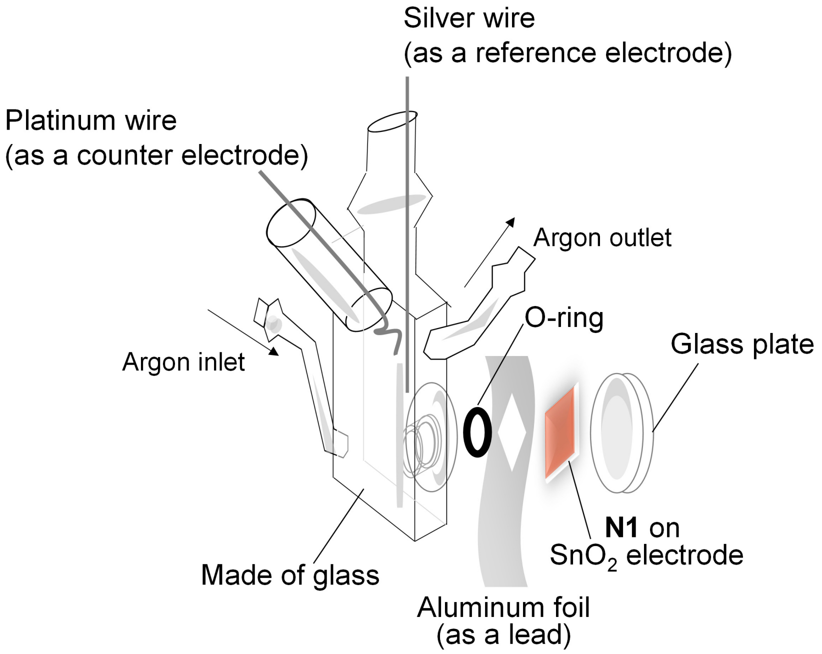

Supplementary Figure 10 | Three-electrode electrochemical cell used for the photocurrent generation. The components are clipped to ensure sealability. The cell is filled with an electrolyte solution during the measurement. The incident light is illuminated in the direction vertical to the SnO_2 electrode.

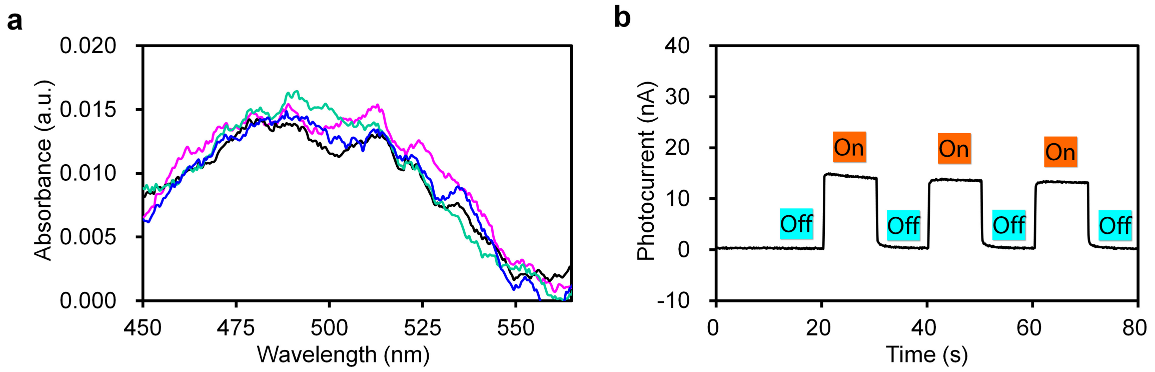

Supplementary Figure 11 | Determination of the quantum yield for the photoelectric conversion of N1. (**a**) Typical absorption spectra of **N1** on a SnO_2 electrode. The data were acquired from four different points. (**b**) Typical anodic current response upon irradiation of the **N1**-physisorbed SnO_2 electrode with intermittent 500-nm light. Light intensity, 0.18 mW. This measurement produced a quantum efficiency of 0.65%.

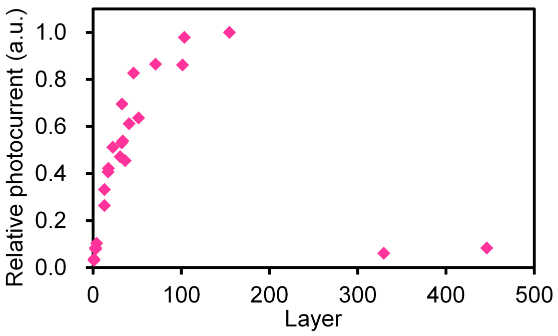

Supplementary Figure 12 | Relationship between the relative photocurrent and thickness of N1 upon irradiation with 500-nm light. The photocurrent was corrected by the intensity of incident light. The highest photocurrent (39 nA with an incident light intensity of 0.16 mW by 154-layer **N1**) is taken as the standard.

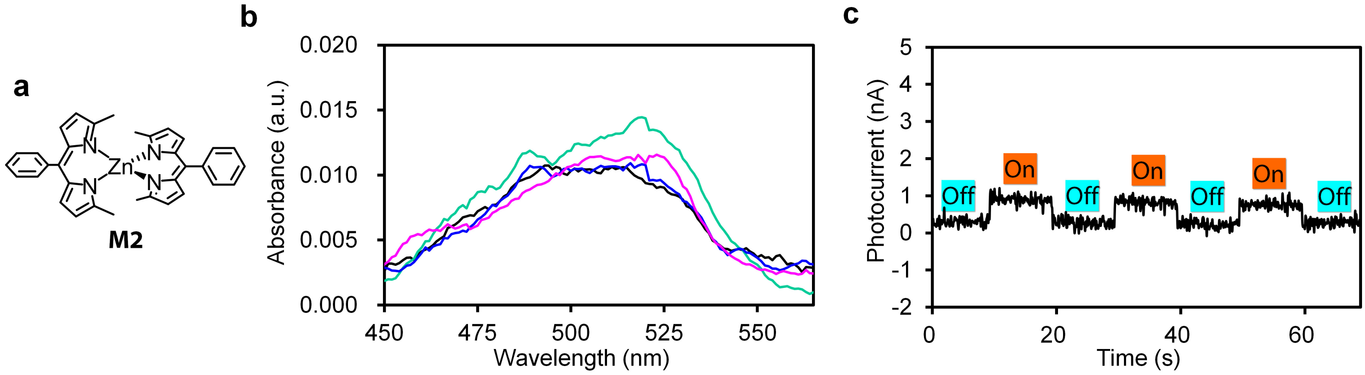

Supplementary Figure 13 | Determination of the quantum yield for the photoelectric conversion of a referential bis(dipyrrinato)zinc(II) complex dye. (**a**) Chemical structure of mononuclear complex **M2**. (**b**) Absorption spectra of **M2** deposited on a SnO_2 electrode. The data were acquired from four different points. (**c**) Anodic current response upon irradiation of the **M2**-physisorbed SnO_2 electrode with intermittent 500-nm light. Light intensity, 0.19 mW. This measurement produced a quantum efficiency of 0.030%.

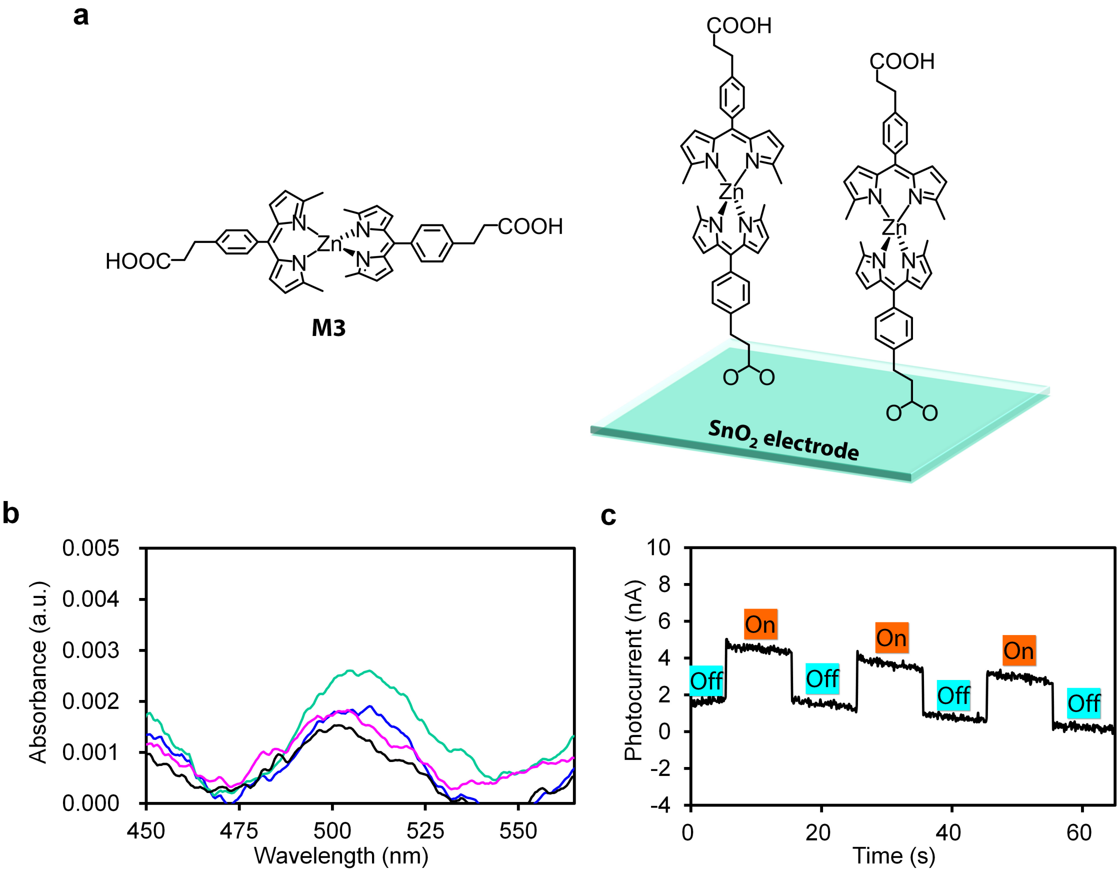

Supplementary Figure 14 | Determination of the quantum yield for the photoelectric conversion of a referential bis(dipyrrinato)zinc(II) complex dye. (**a**) Chemical structure of mononuclear complex **M3** and a schematic illustration on its self-assembled monolayer (SAM) on a SnO_2 electrode. (**b**) Absorption spectra of **M3** immobilized as a SAM on a SnO_2 electrode. The data were acquired from four different points. (**c**) Anodic current response upon irradiation of the **M3**-chemisorbed SnO_2 electrode with intermittent 500-nm light. Light intensity, 1.8 mW. This measurement produced a quantum efficiency of 0.069%.

Supplementary References

1. Tkatchenko, A. Commensurate monolayers on surfaces: Geometry and ground states. *Phys. Rev. B* **75,** 235411 (2007).
2. Kambe, T. *et al.* π-Conjugated nickel bisdithiolene complex nanosheet. *J. Am. Chem. Soc.* **135,** 2462–2465 (2013).
